# Supplementary material for: A Prospective Study on Deep Inspiration Breath Hold Thoracic Radiation Therapy Guided by Bronchoscopically Implanted Electromagnetic Transponders
Source: Cancers (Basel). 2024 Apr 17;16(8):1534. doi: 10.3390/cancers16081534 (PMC11048337; doi:10.3390/cancers16081534)
Supplement: Supplementary file 1 [file cancers-16-01534-s001.zip › cancers-2914826-supplementary.pdf]

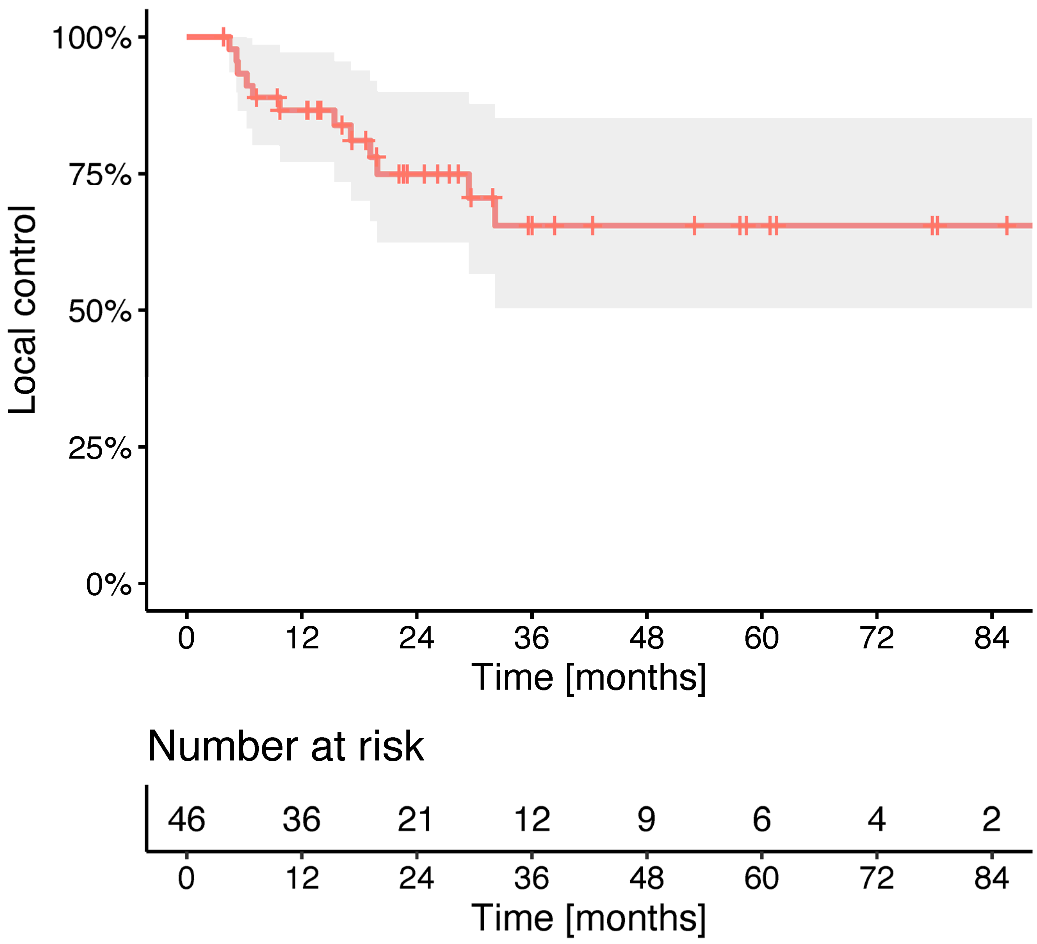
Local control
100%
75%
50%
25%
0%
0
12
24
36
48
60
72
84
Time [months]
Number at risk
46
36
21
12
9
6
4
2
0
12
24
36
48
60
72
84
Time [months]
(a)

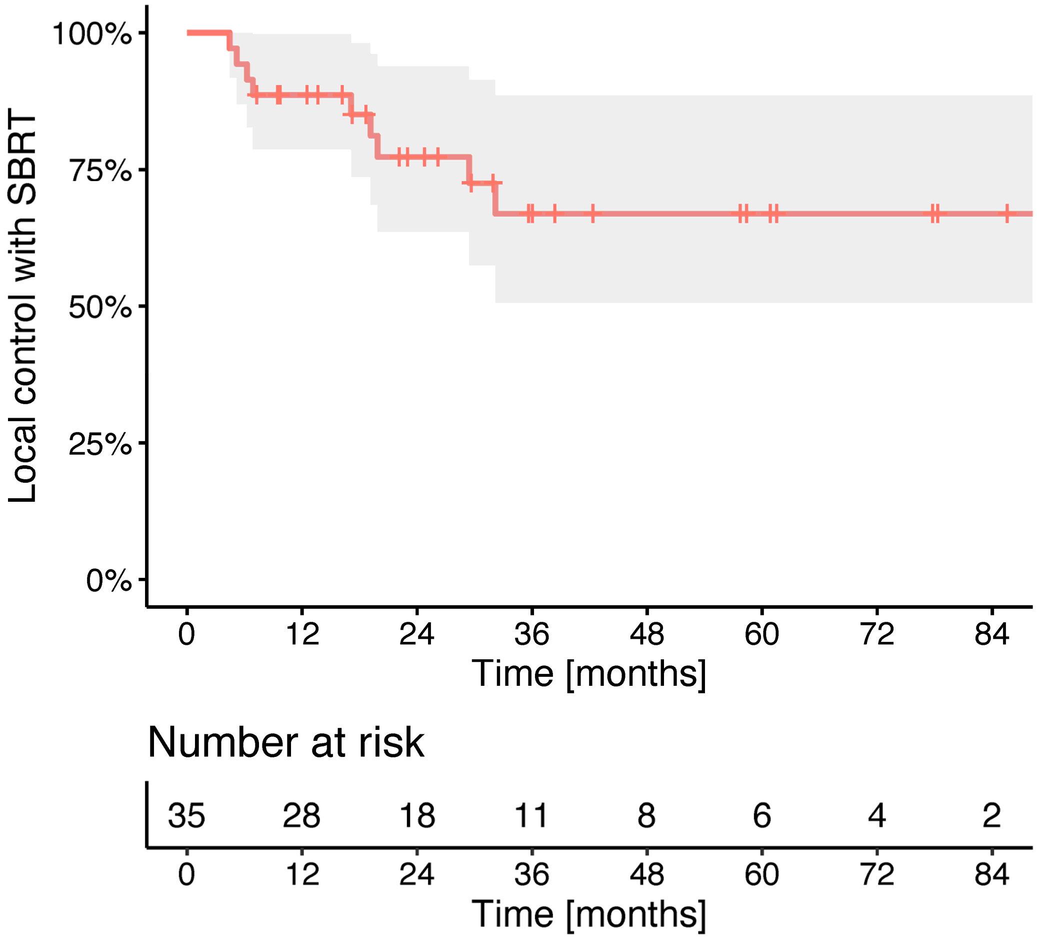

(**b**)

Figure S1. Kaplan–Meier plots of local control for (a) all patients treated with EMT-guided RT and (b) patients treated with EMT-guided stereotactic body RT (SBRT).
